# Comprehensive analysis to identify the influences of SARS-CoV-2 infections to inflammatory bowel disease

**DOI:** 10.3389/fimmu.2023.1024041

**Published:** 2023-02-03

**Authors:** Chengyan Zhang, Zeyu Ma, Xi Nan, Wenhui Wang, Xianchang Zeng, Jinming Chen, Zhijian Cai, Jianli Wang

**Affiliations:** ^1^ Bone Marrow Transplantation Center of the First Affiliated Hospital, Zhejiang University School of Medicine, Hangzhou, China; ^2^ Institute of Immunology, Zhejiang University School of Medicine, Hangzhou, China; ^3^ Department of Anorectal, Affiliated Hangzhou Dermatology Hospital, Zhejiang University School of Medicine, Hangzhou, China; ^4^ Department of Orthopaedics of the Second Affiliated Hospital, Zhejiang University School of Medicine, Hangzhou, China; ^5^ Institute of Hematology, Zhejiang University, Hangzhou, China; ^6^ Zhejiang Engineering Laboratory for Stem Cell and Immunotherapy, Hangzhou, China

**Keywords:** IBD, COVID-19, hub genes, differentially expressed genes, single cell atlas, inflammation-related genes

## Abstract

**Background:**

Coronavirus disease 2019 (COVID-19) and inflammatory bowel disease (IBD) are both caused by a disordered immune response and have direct and profound impacts on health care services. In this study, we implemented transcriptomic and single-cell analysis to detect common molecular and cellular intersections between COVID-19 and IBD that help understand the linkage of COVID-19 to the IBD patients.

**Methods:**

Four RNA-sequencing datasets (GSE147507, GSE126124, GSE9686 and GSE36807) from Gene Expression Omnibus (GEO) database are extracted to detect mutual differentially expressed genes (DEGs) for IBD patients with the infection of severe acute respiratory syndrome coronavirus 2 (SARS-CoV-2) to find shared pathways, candidate drugs, hub genes and regulatory networks. Two single-cell RNA sequencing (scRNA-eq) datasets (GSE150728, PRJCA003980) are used to analyze the immune characteristics of hub genes and the proportion of immune cell types, so as to find common immune responses between COVID-19 and IBD.

**Results:**

A total of 121 common DEGs were identified among four RNA-seq datasets, and were all involved in the functional enrichment analysis related to inflammation and immune response. Transcription factors-DEGs interactions, miRNAs-DEGs coregulatory networks, and protein-drug interactions were identified based on these datasets. Protein-protein interactions (PPIs) was built and 59 hub genes were identified. Moreover, scRNA-seq of peripheral blood monocyte cells (PBMCs) from COVID-19 patients revealed a significant increase in the proportion of CD14^+^ monocytes, in which 38 of 59 hub genes were highly enriched. These genes, encoding inflammatory cytokines, were also highly expressed in inflammatory macrophages (IMacrophage) of intestinal tissues of IBD patients.

**Conclusions:**

We conclude that COVID-19 may promote the progression of IBD through cytokine storms. The candidate drugs and DEGs-regulated networks may suggest effective therapeutic methods for both COVID-19 and IBD.

## 1 Introduction

Coronavirus disease 2019 (COVID-19), caused by severe acute respiratory syndrome coronavirus 2 (SARS-CoV-2), has spread rapidly since December 2019 and led to millions of confirmed and dead cases worldwide (1). Severity and mortality of COVID-19 are identified to associate with age, cardiovascular disease, chronic pulmonary disease, obesity, diabetes, and cancer (2, 3). As the COVID-19 pandemic expands, the public has shown increasing concern regarding the influence of COVID-19 on patients with those chronic conditions.

Inflammatory bowel disease (IBD) is a chronic, relapsing, and incurable inflammatory condition of gastrointestinal tract with autoimmune disorders, and comprises two distinct forms: Crohn’s disease (CD) and Ulcerative colitis (UC) (4–6). The prevalence of IBD has rapidly risen in every continent and the globalization of IBD has posed great challenges on the health-care system (7, 8). Patients with IBD require treatment with immunosuppressive and immunomodulatory agents, which lead to increased risk of bacterial or viral infections (9, 10). Nevertheless, the incidence of COVID-19 among IBD patients appears to be comparable to that of the general population based on the real-world evidence (11). According to the Surveillance Epidemiology of Coronavirus Under Research Exclusion for inflammatory Bowel Disease (SECURE-IBD) database, an international registry of IBD patients infected with SARS-CoV-2, it has been reported that corticosteroids and mesalamine, rather than tumor necrosis factor-α (TNF-α) antagonists are associated with an increased risk of COVID-19 in IBD patients (12, 13). Other risk factors of developing COVID-19 include active disease status, and elderly patients with IBD comorbidities (14). However, so far, little is known about the impact of COVID-19 on the severity and progression of IBD.

**Figure 1:**
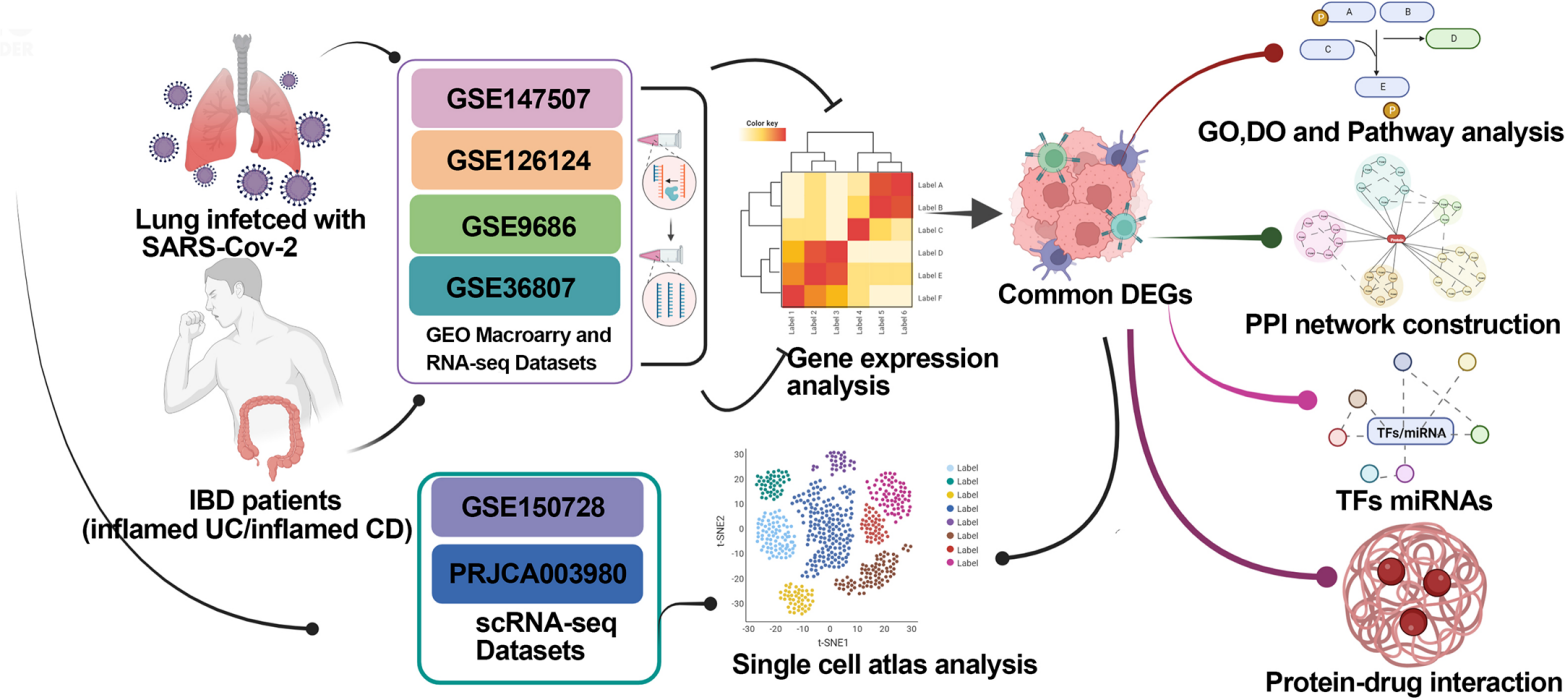
Schematic diagram of the whole workflow of this study.

In this study, we screened the molecular and cellular intersections between COVID-19 and IBD-associated gene expression using transcriptome and single cell sequencing data. Our work identified the promotion to the IBD severity by COVID-19 *via* excessive cytokine storm, and may help with the understanding and medication management in IBD patients infected with SARS-CoV-2. Schematic diagram of the whole workflow in this study is presented in **Figure 1**

## 2 Materials and methods

### 2.1 Collection of the RNA sequencing data

The transcriptional profiling of lung biopsies and cells infected by SARS-CoV-2 (GSE147507) were obtained from the GEO database, and the expression profile consisting of the GSE147507 dataset was processed using the Illumina NextSeq 500 (15). Similarly, the CD dataset (GSE126124), which contained 37 CD patients and 21 healthy controls were analyzed using the platform of GPL6244 Affymetrix Human Gene 1.0 ST Array (16). The other two gene expression profiles, the UC datasets (GSE9686 and GSE36807) containing 20 UC patients and 15 healthy controls were analyzed using the GPL5760 Affymetrix GeneChip Human Genome U133 Plus 2.0 Array (17) and GPL570 Affymetrix Human Genome U133 Plus 2.0 Array (18), respectively. For the validation of our findings, GSE157103 and GSE75214 datasets were downloaded and analyzed. GSE157103 dataset (including 100 COVID-19 samples and 26 non-COVID-19 samples) was based on Illumina NovaSeq 6000 platform (19). The GSE75214 datasets, containing 8 active CD patients, 74 active UC patients, 23 inactive UC patients and 11 healthy controls, was analyzed using the Affymetrix GPL6244 platform (20). The gene expression matrix files for the data from all these databases were derived from raw transcriptome data using Perl.

### 2.2 Identification of DEGs and the common DEGs among COVID-19 and IBD

To acquire DEGs for the datasets GSE147507 and GSE126124, the “limma” package (21) was employed to select DEGs with |log_2_FC | > 0.585 (22) and p-value < 0.05 (23). For UC dataset, we firstly extracted and integrated GSE9686 and GSE36807 datasets, then normalized the data by using “merge” function in R and ‘limma’ package, and finally screened the DEGs with |log_2_FC | > 0.585 and p-value < 0.05 using ‘limma’ package. The common DEGs among COVID-19 and IBD (including CD and UC) were gained using Jvenn, an online Venn analysis tool (24).

### 2.3 Enrichment analysis of GO ontology, disease ontology and pathway

In order to further explore the potential biological functions of these common DEGs and their correlation with diseases, the clusterprofiler package (25) was used to analyze Gene Ontology (GO) and Disease Ontology (DO), as well as enrichment analysis of these common DEGs at a threshold p-value of < 0.05. In addition, the Enrichr, an online tool (https://maayanlab.cloud/Enrichr/), was used to identify the significant signalling pathway of these shared DEGs, including Kyoto Encyclopaedia of Genes and Genomes (KEGG) Pathways, WikiPathways, Reactome, and Hallmark. The statistical significance cut-off was set at p-value < 0.05.

### 2.4 Construction of PPI networks and regulatory networks of the common DEGs

The protein-protein interaction (PPI) network of these common DEGs were obtained from the STRING database (https://string-db.org/) and gene interactions with a combined score of ≥ 0.4 were chosen to construct the PPI networks which were visualized using Cytoscape (v3.8.2). The degree of each node was calculated using the Cytoscape plugin CytoHubba, which is an important application for assessing and extracting the centrum of biological networks (26), and those with a degree score of > 10 were identified as hub genes (27).

To further explore the transcriptional factors (TFs) regulating common DEGs, we used the ‘NetworkAnalyst’ function to discover topologically plausible TFs obtained from the JASPAR database that trend to bind to our common DEGs. JASPAR database is an open access database of transcription factor binding profiles, including six different taxonomic groups (28). NetworkAnalyst is used for meta-analysis of gene expression data on the basis of PPI network to further investigate biological mechanisms and roles (29). Besides, interaction analysis of target gene-miRNA were performed to identify miRNAs which bind to transcripts of common DEGs to affect protein expressions. Here, we used the ‘NetworkAnalyst’ function to detect miRNAs gained from MiRTarBase (30), a database of miRNAs-target gene interactions. We used the Cytoscape to visualize the interaction network of TFs–genes and miRNAs–genes.

### 2.5 Processing and analysing the scRNA-seq data

The single cell RNA sequencing (scRNA-seq) data (31) (GSE150728) of PBMCs from 7 COVID-19 patients and 6 healthy controls was obtained from the GEO database. Meanwhile, the scRNA-seq data (32) (the accession number BioProject: PRJCA003980) of CD45^+^ mucosal immune cells from 12 IBD (including 6 CD and 6 UC) and 3 healthy controls was downloaded from the GSA database. The Seurat (version 4.1.1) package was performed to process the raw count matrix (UMI counts of each sample). Low quality cells (< 200 genes/cell, < 10 cells/gene and > 15% mitochondrial genes) were excluded. Subsequently, normalization was performed using the ‘NormalizeDate’ function based on the corrected expression matrix. Cell clustering and dimensional reduction were conducted using Seurat package based on the corrected expression data (33). The ‘FindVariableGenes’ function was performed to scale the data based on the expression of 2000 highly variable genes (HVGs). Principal component analysis of these HVGs was performed by the ‘RunPCA’ function (34), and the batch effects between different samples were eliminated using ‘harmony’ function (35). The resolution was set to 1.0 to determine cell types in all cell populations. The aggregated cells were then projected into a two-dimensional space using the ‘RunTSNE’ function, and the ‘Dimplot’ function was performed to visualize the cell clusters (32), which were finally annotated according to known markers.

### 2.6 Comparing the expression of IRGs in IBD patients before and after infliximab treatment

To further understand the pathogenesis of inflammation-related DEGs (IRGs) identified in scRNA-seq data, we downloaded RNA-seq data (GSE16879) of IBD patients before and after the first infliximab (TNF-α antagonists) treatment from the GEO database. The dataset included 12 normal controls, 37 CD samples before treatment and 31 CD samples after treatment, as well as 24 UC samples before treatment and 21 UC samples after treatment using the platform of GPL570 Affymetrix Human Genome U133 Plus 2.0 Array. The ‘heatmap’ package was performed to display the differences in the expression of IRGs in IBD patients before and after the first infliximab treatment.

### 2.7 Evaluation of applicant drugs

The evaluation of protein-drug interactions (PDIs) is very important for understanding disease characteristics, thus we used Enrichr, an online tool, to predict PDIs. Drug molecules were identified using the Drug Signatures database (DSigDB) *via* Enrichr based on the common DEGs. DSigDB (36) is the data source for recognizing targeted drug substances linked to DEGs.

### 2.8 Statistical analyses

The variations in both groups were assessed utilizing an unpaired Student’s t-test, and variations in multiple groups were assessed by means of one-way or two-way ANOVA. The statistical significance cut-off was set at p-value < 0.05.

## 3 Results

### 3.1 Identification of the common DEGs between COVID-19 and IBD

**Figure 2:**
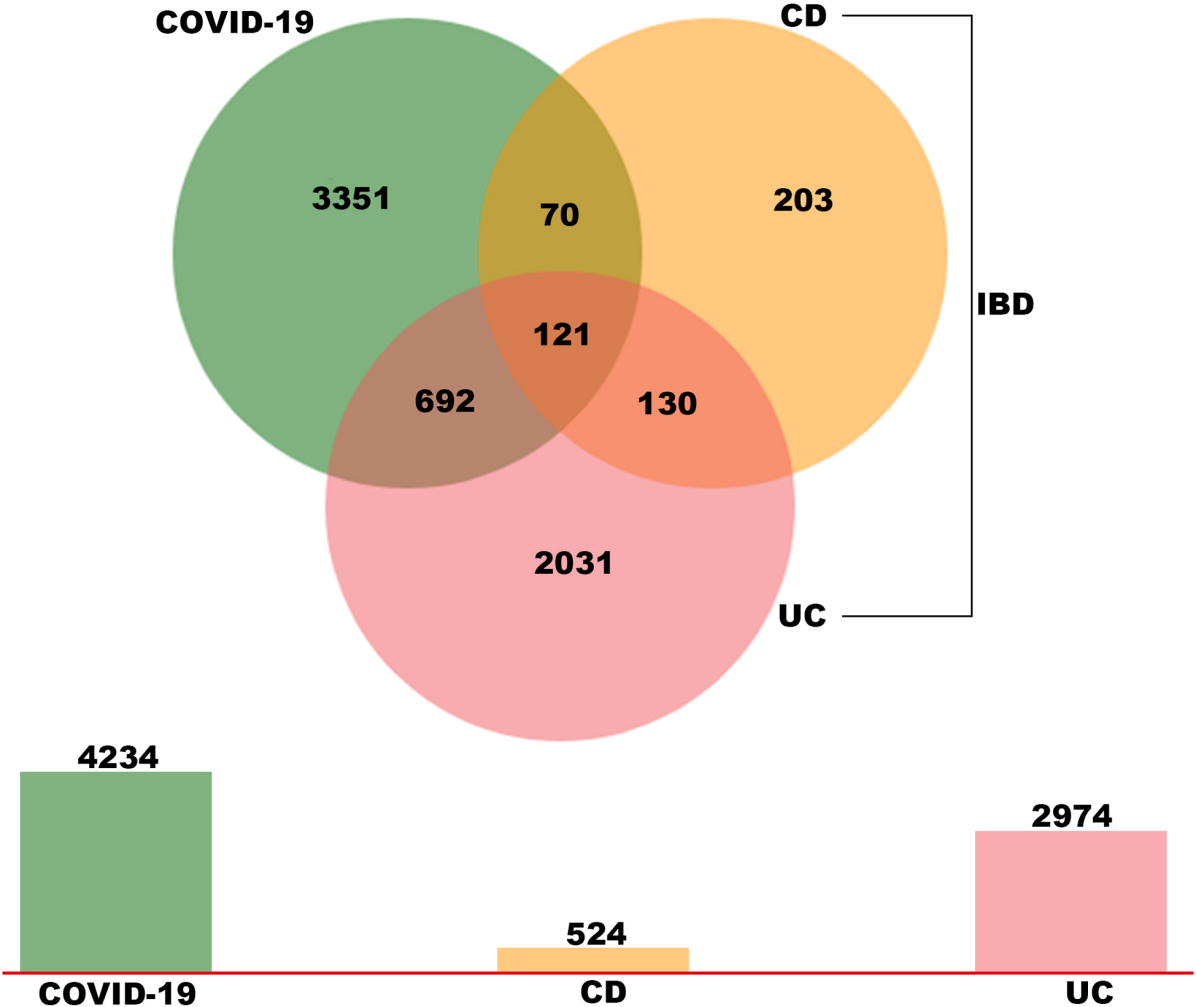
Identification of common DEGs between COVID-19, UC and CD *via* a Venn diagram. This study incorporates four RNA-sea datasets comprising of COVID-19 (GSE147507), UC (GSE 9686 and GSE 36807) and CD (GSE 126124). A total of 121 common DEGs were identified among COVID-19, UC and CD by integrated analysis.

To explore the potential interrelationships and implications of COVID-19 and IBD patients, the human RNA-seq dataset GSE147507 was carried out to identify DEGs for COVID-19 patients. Meanwhile, GSE126124, GSE9686 and GSE36807 human RNA-seq dataset were used to investigate DEGs for IBD patients (including CD and UC). Firstly, 4234 DEGs were screened from COVID-19 samples, including 2267 up-regulated genes and 1967 down-regulated genes. Similarly, 524 DEGs (327 up-regulated and 197 down-regulated) were screened in the CD cohort and 2974 DEGs (2091 up-regulated and 883 down-regulated) were obtained in the UC cohort. We identified 121 common DEGs between these datasets through a Venn diagram (**Figure 2** and **Supplementary Table S1**). Thus, the two diseases (COVID-19 and IBD) are related together because of the common DEGs they share with each other. The common DEGs were employed to accomplish further exploration.

### 3.2 GO, DO and pathway enrichment analysis of common DEGs

**Figure 3:**
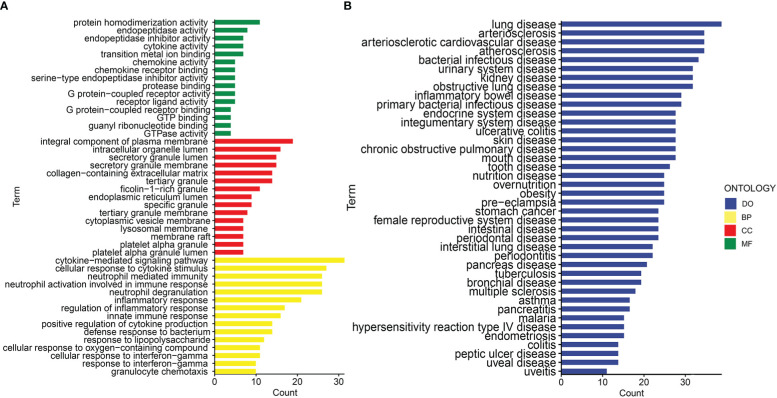
The bar graphs of the ontological enrichment analysis of the shared DEGs between COVID-19 and IBD. **(A)** Gene Ontology (GO) enrichment analysis of the shared DEGs. **(B)** Disease Ontology (DO) enrichment analysis of the shared DEGs.

**Figure 4:**
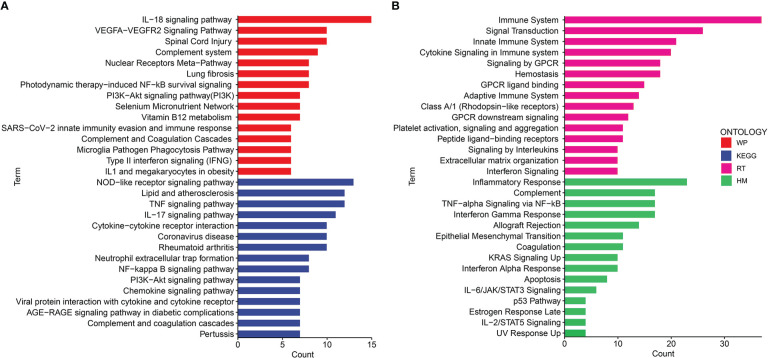
The bar graphs of the pathway enrichment analysis of the shared DEGs between COVID-19 and IBD. **(A)** WikiPathways and KEGG 2019 human Pathways **(B)** Biocarta pathways and Hallmark.

Gene ontology (GO) and disease ontology (DO) enrichment analysis were carried out to investigate the potential biological functions of shared DEGs through clusterProfile package. The GO analysis showed that common DEGs were significantly involved in cytokine-mediated signaling pathway, neutrophil mediated immunity, and inflammatory response; protein homodimerization activity, endopeptidase activity and cytokine activity; integral component of plasma membrane, intracellular organelle lumen and secretory granule lumen (**Figure 3A** and **Supplementary Table S2**). Meanwhile, the DO analysis revealed that common DEGs were markedly enriched in lung disease, atherosclerosis, obstructive lung disease, kidney disease, inflammatory bowel disease and ulcerative colitis (**Figure 3B** and **Supplementary Table S3**). The most impacted pathways of common DEGs among COVID-19 and IBD were gathered from four global databases, including KEGG, WikiPathways, Reactome and Hallmark. The pathway enrichment analysis revealed that shared DEGs were involved in the IL-18 signaling pathway, PI_3_K-Akt signaling pathway, TNF signaling pathway, IL-17 signaling pathway, cytokine-cytokine receptor interaction, cytokine signaling in immune system and inflammatory response (**Figures 4A, B** and **Supplementary Table S4-S5**). These findings suggested that common DGEs were all involved in the functional enrichment related to inflammation and immune response.

### 3.3 Identification of 59 hub genes and construction of regulatory networks

To anticipate the interactions and adhesion pathways of shared DEGs, we constructed PPI networks from STRING database and visualized them in Cytoscape. The PPI network of common DEGs consists of 121 nodes and 703 edges, and was displayed in **Supplementary Figure 1A**. Then, 59 hub genes were identified with a threshold degree of >10 using Cytohubba plugin among 121 common DEGs (**Supplementary Figure 1B**, **Supplementary Figure 2** and **Supplementary Table S6**). Besides, to discover key molecules regulating common DEGs, a network-based approach was adopted to interpret the regulatory transcription factors (TFs) and miRNAs. The TF regulators network of shared DEGs was visualized in **Supplementary Figure 3A**, and the interaction between miRNAs and shared DEGs was shown in **Supplementary Figure 3B**. We found a total of 59 TFs and 69 post-transcriptional (miRNAs) regulatory signals that regulated these hub genes, indicating a strong interference between them.

### 3.4 Immune landscape of hub genes in COVID-19 single cell atlas

**Figure 5:**
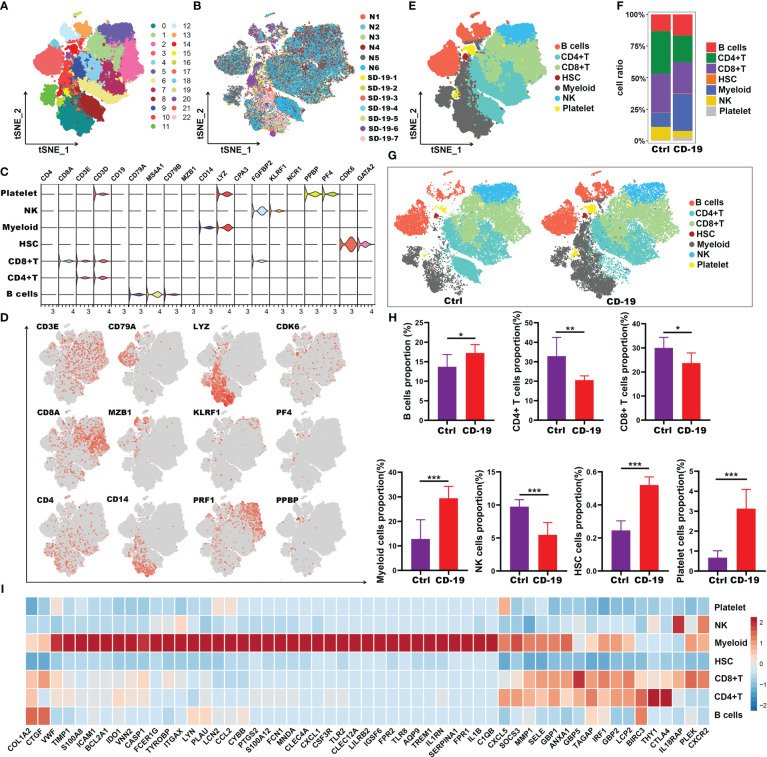
Immune characteristics of the hub genes at the single-cell resolution in PBMCs of COVID-19. **(A, B)** t-SNE plot displaying 63,314 immune cells from 13 samples; color denotes cluster origin **(A)** and sample origin **(B)**. **(C)** Violin plots showing expression levels of specific markers for platelets, NK, myeloid cells, HSC, CD8^+^ T, CD4^+^ T and B cells. **(D)** t-SNE plots demonstrating the expression of selected marker genes in different cell types. **(E)** t-SNE plot representation of the major cell types of COVID-19 PBMCs. **(F)** Proportion of each cell types in COVID-19 and healthy individuals. **(G)** t-SNE plot representation of each cell types in COVID-19 and healthy individuals. **(H)** Proportion of each cell types between COVID-19 and healthy controls. **(I)** Heatmap showing expression patterns of hub genes in each cell types. *p < 0.05; **p < 0.01; ***p < 0.001; ns, not significant.

To evaluate the immune landscape of these hub genes in COVID-19 patients, scRNA-seq dataset GSE150728 of PBMCs from 7 COVID-19 patients and 6 healthy controls was used. A total of 61,772 cells were filtered and split into 23 clusters using unsupervised graph-based clustering (**Figure 5A**). The t-Stochastic Neighborhood Embedding (t-SNE) plot displaying 61,772 immune cells from 13 samples was visualized in **Figure 5B**. We subsequently identified seven major lineages (B cells, CD4^+^T, CD8^+^T, HSC, Myeloid, NK and platelet cells) based on canonical cell markers (**Figures 5C-E**). Dimensionality reduction displayed significant phenotypic difference between patients with COVID-19 and controls (**Figures 5F, G**). We then quantified the changes in the proportion of cell types. Compared with healthy controls, the relative proportion of these cell types in COVID-19 patients showed an increase in myeloid cells, HSC, platelet and B cells, and a decrease in CD4^+^T, CD8^+^ T and NK cells (**Figure 5H**). Next, to explore the immune characteristics of hub genes in COVID-19 patients, we further analyzed the expression of these genes in seven major group of cells. Among 59 hub genes, there were 38 genes highly expressed in myeloid cells, compared with other cell types (**Figure 5I**). Thus, these results indicated that the 38 genes may play an important role in myeloid cells and thus have a great impact on the occurrence and development of COVID-19 disease.

### 3.5 High expression of hub genes in CD14^+^ monocytes of COVID-19 patients

**Figure 6:**
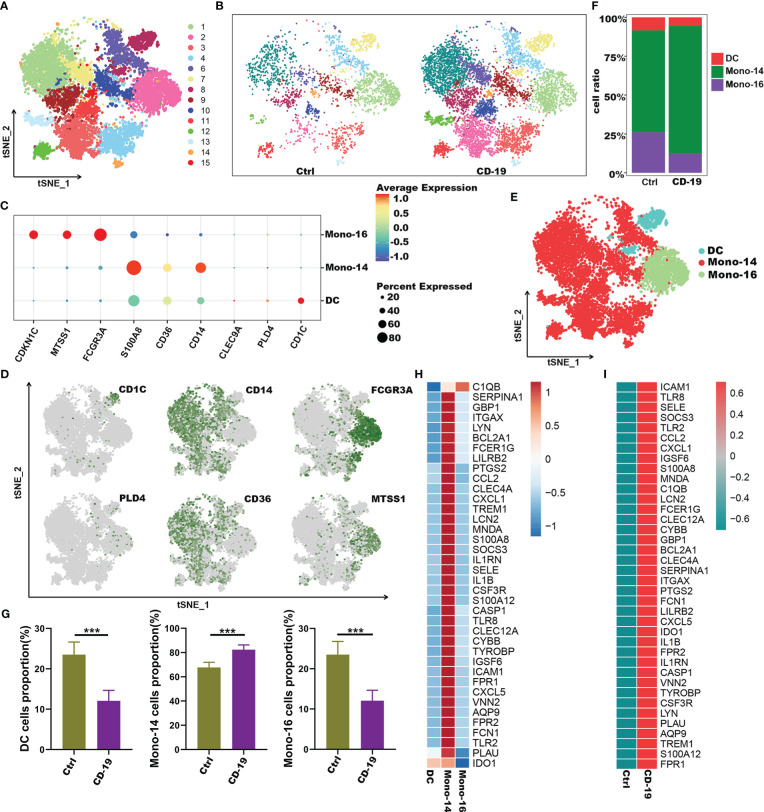
Immune characteristics of the hub genes at the single-cell atlas in myeloid cell metaclusters with COVID-19. **(A)** t-SNE plot visualizing the clusters of myeloid cells. **(B)** t-SNE plot visualization of the clusters between COVID-19 and healthy controls. **(C)** Dot plot depicting the expression level of significant marker genes. **(D)** t-SNE plot of marker genes enriched in subpopulations of myeloid cells. **(E)** t-SNE plot of subgroups of myeloid cells in COVID-19. **(F)** Proportion of subgroups of myeloid cells in COVID-19 and healthy controls. **(G)** Bar plots showing the relative frequencies of different myeloid cells subgroups in COVID-19 and healthy controls. **(H)** Heatmap showing gene expression patterns of hub genes in subpopulations of myeloid cells. **(I)** Heatmap showing expression patterns of hub genes in CD14^+^monocyte cells between COVID-19 and healthy controls. ***p < 0.001; ns, not significant.

To further explore the potential role of these 38 genes, we extracted myeloid cells for subgroup analysis and explored the distribution of 38 genes in the subgroups of myeloid cells. Here, we performed unsupervised graph-based clustering on myeloid cells, which identified 15 clusters (**Figure 6A**). The distribution of 15 clusters between COVID-19 patients and controls was visualized in t-SNE plot (**Figure 6B**). The clustering of the myeloid cells emerges 3 sub-populations with specific gene signatures, including dendritic cells (DC), CD14^+^ monocytes and CD16^+^ monocytes (**Figures 6C-E**). Meanwhile, we quantified the changes in the proportion of three different cell types. We observed a decrease in the number of DCs and CD16^+^ monocytes, as well as a significant increase in the proportion of CD14^+^ monocytes in COVID-19 patients (**Figures 6F, G**). Interestingly, we noticed that almost all these 38 genes were highly expressed in CD14^+^ monocytes compared with the other two types of cells except *C1qb* gene (**Figure 6H**). Of note, in contrast to the healthy controls, these 38 genes were highly expressed in CD14^+^ monocytes of COVID-19 patients (**Figure 6I**). Therefore, our data indicated that genes such as *Il1b*, *S100a*, *Il1rn* and other inflammatory factors which were highly expressed by CD14^+^ monocytes, may be the main contributors to the cytokine storm in COVID-19.

### 3.6 Immune characteristics of the 38 IRGs in IBD single cell atlas

**Figure 7:**
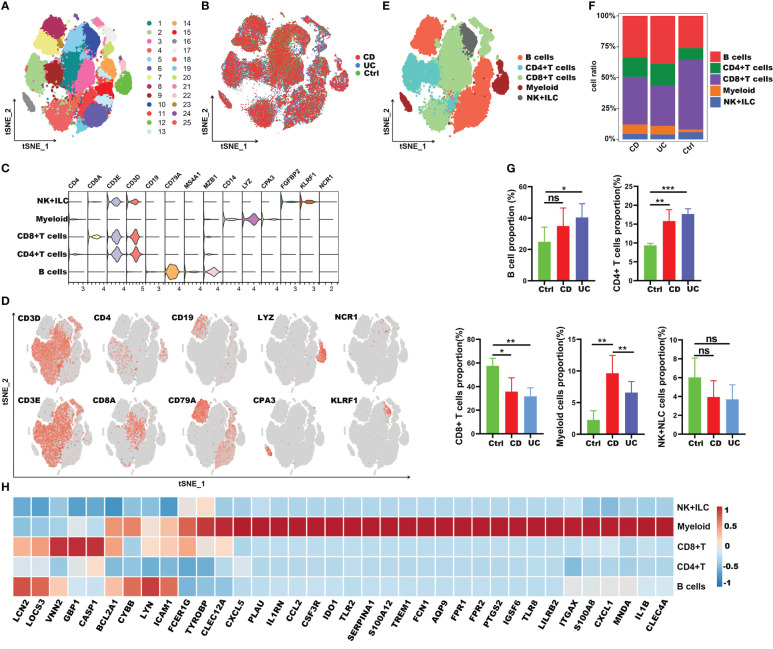
Immune landscape of the IRGs at the single-cell resolution in CD45^+^ immune cells of IBD. **(A, B)** t-SNE plot displaying 63,314 immune cells in IBD and healthy controls; color denotes cluster origin **(A)** and sample origin **(B)**. **(C)** Violin plots showing expression levels of specific markers for NK+ILC, myeloid cells, CD8^+^ T and CD4^+^ T cells. **(D)** t-SNE plot demonstrating the expression of marker genes in different cell types. **(E)** t-SNE plot representation of the major cell types of CD45^+^ immune cells in IBD patients. **(F)** Proportion of each cell types in IBD patients and healthy controls. **(G)** Bar plots showing the relative frequencies of each cell types in IBD and healthy controls. **(H)** Heatmap showing the expression patterns of IRGs in major cell types. *p < 0.05; **p < 0.01; ***p < 0.001; ns, not significant.

To clarify the impact of COVID-19 on the process of IBD, we subsequently explored the immune characteristics of these 38 inflammation-related genes (IRGs) in IBD patients. Here, the scRNA-seq dataset of 12 IBD patients (including 6 CD and 6 UC) and 3 healthy controls was obtained from the article published by Professor JinJin (32). A total of 63,314 CD45^+^ immune cells were obtained and split into 25 clusters using unsupervised graph-based clustering (**Figures 7A, B**). We further partitioned these cells into five major lineages (including CD4^+^ T cells, CD8^+^ T cells, B cells, myeloid cells, natural killer (NK) cells + innate lymphoid cells (ILCs)) according to canonical cell markers (**Figures 7C-E**). Compared with healthy controls, the intestinal tissues of IBD patients were infiltrated with a greater amount of B cells, CD4^+^ T cells and myeloid cells, though there were fewer CD8^+^ T cells and comparable number of NK+ILC cells (**Figures 7F, G**). We then analyzed the expression of IRGs in different cell types. Interestingly, the results were consistent with those in COVID-19; 27 of the IRGs were highly expressed in myeloid cells, except *Icam1, Lyn, Cybb, Casp1*, *Gbp1, Vnn2, Socs3, Bcl2a1* and *Lcn2* genes (**Figure 7H**). As a result, these findings suggested that the 27 common IRGs may also play an important part in the process of intestinal inflammation in IBD patients.

### 3.7 Accumulation of IRGs in inflammatory macrophages of IBD patients

**Figure 8:**
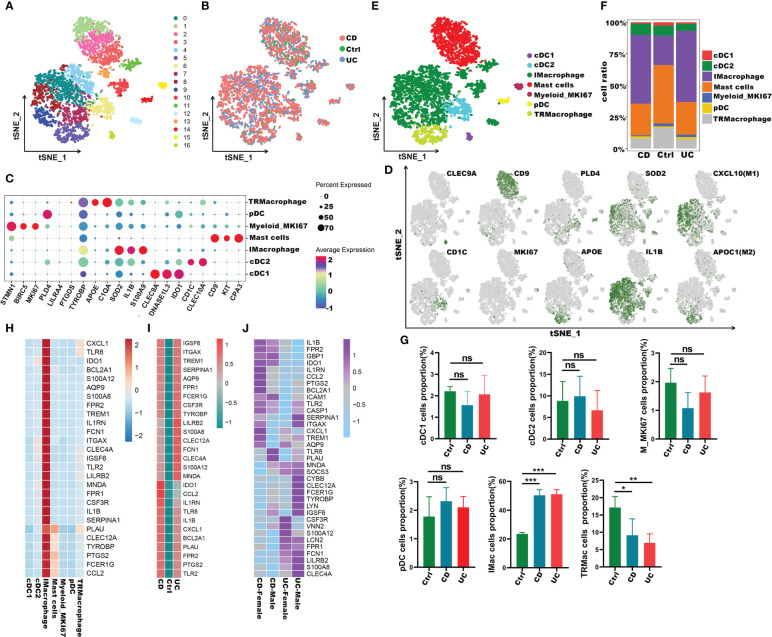
Immune characteristics of the IRGs at the single-cell resolution in myeloid cells of IBD. **(A, B)** t-SNE plot visualizing the clusters of myeloid cells from IBD and healthy controls; color denotes cluster origin **(A)** and samples origin **(B)**. **(C)**Dot plot depicting the gene expression level of significant marker genes in subgroups of myeloid cells. **(D)** t-SNE plot visualizing the expression of marker genes in different subgroups of myeloid cells. **(E)** t-SNE plot of subgroups of myeloid cells of IBD patients. **(F)** Proportion of subgroups of myeloid cells in IBD patients and health controls. **(G)** Bar plots showing the relative frequencies of different subgroups of myeloid cells in IBD and healthy controls. **(H)** Heatmap displaying the expression patterns of the IRGs in subgroups of myeloid cells. **(I)** Heatmap showing expression patterns of IRGs in IMacrophages between IBD and healthy individuals. **(J)** Heatmap showing expression patterns of IRGs in UC and CD patients with different genders *p < 0.05;**p < 0.01; ***p < 0.001; ns, not significant.

**Figure 9:**
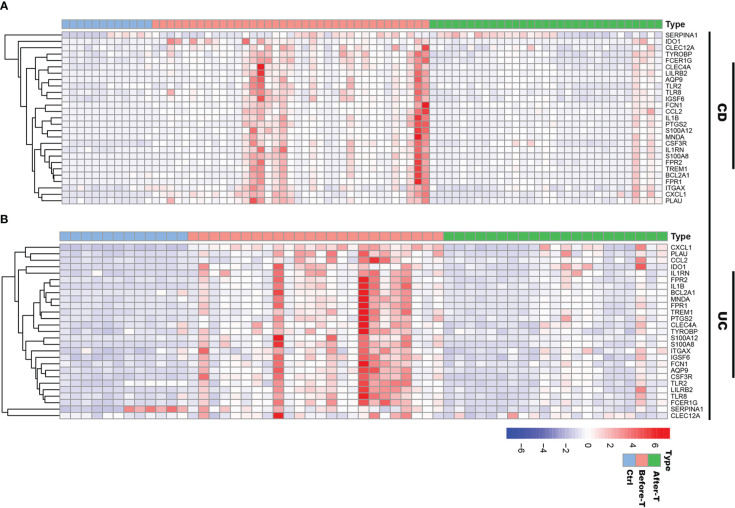
The expression profiles of IRGs in IBD patients before and after infliximab treatment. **(A)** Heatmap of the IRGs expression before and after infliximab treatment in CD patients, compared with healthy controls. **(B)** Heatmap of the IRGs expression before and after infliximab treatment in UC patients, compared with healthy controls.

To understand the role of these common 27 IRGs in myeloid cells of IBD patients, the myeloid cells were further clustered into 17 clusters (**Figure 8A**). We visualized data by dimensionality reduction using t-Stochastic Neighborhood Embedding (t-SNE), as shown in **Figure 8B**. The t-SNE plot identified seven subtypes of myeloid cells, which were characterized by the expression of cell markers *Clec9a, Cd1c, Cd9, Mki67, Pld4, Apoe, Sod2, Il1b* (**Figures 8C-E**). We also quantified the changes in the proportion of cell types between IBD and control samples. Compared with healthy controls, inflammatory macrophages (IMacrophages) were more abundant in CD and UC patients, while tumor-related macrophages (TRMacrophages) were less infiltrated (**Figures 8F, G**). Most types of cells had no significant difference between IBD patients and healthy controls, including cDC1 cells, cDC2 cells, Mki67^+^ myeloid cells, and pDC cells (**Figures 8F, G**). We then analyzed the expression of 27 IRGs highly expressed in myeloid cells, and found that these genes were accumulated in IMacrophages (**Figure 8H**). Compared with healthy controls, these IRGs also had a higher expression in the IMacrophages of UC and CD patients (**Figure 8I**). Interestingly, the expression profiles of these IRGs were different between UC and DC. IRGs such as *Ido1, Ccl2, Il1rn, Tlr8, Cxcl1, Bcl2a1, Plau, Fpr2, Ptgs2* and *Tlr2* were more abundant in CD, especially in female CD; while others were more abundant in UC, especially in male UC (**Figures 8I, J**). To further understand the pathogenesis of these IRGs, the GO, DO and KEGG analysis of these IRGs were performed. Consistent with the previous analysis results, these IRGs were enriched in cytokine-mediated signaling pathway, neutrophil mediated immunity, and inflammatory response; lung disease, inflammatory bowel disease and ulcerative colitis (**Supplementary Table S7-S8**). Subsequently, we further verified the expression of these IRGs in diseases using other GEO datasets (**Supplementary Figure 4A, B**). Also, we compared the differences of the expression of IRGs in IBD patients before and after the first infliximab treatment. Of note, the results showed that the expression level of most IRGs decreased significantly after IBD treatment (**Figures 9A, B**). To conclude, these results indicated that the overexpression of IRGs, which are highly expressed in PBMCs of COVID-19 patients, may also promote the progression of IBD through cytokine storms.

### 3.8 Identification of candidate drugs

Assessing protein drug interactions is important for understanding disease characteristics (37). In the aspects of common DEGs as potential drug targets in COVID-19 and IBD, we identified 10 possible chemical compounds using the Drug Signatures database (DSigDB) *via* Enrichr. The top 10 compounds were extracted according to their p values (**Table 1**). These potential drugs are suggested for the common DEGs, and can be common chemical compounds for both diseases.

**Table 1:** List of the top 10 potential chemical compounds for COVID-19 and IBD.

Name	FDR	Chemical Formula
estradiol CTD 00005920	4.67E-09	C18H24O2
Retinoic acid CTD 00006918	6.60E-09	C20H28O2
progesterone CTD 00006624	5.77E-13	C21H30O2
raloxifene CTD 00007367	1.10E-06	C28H27NO4S
calcitriol CTD 00005558	7.11E-05	C27H44O3
(-)-Epigallocatechin gallate CTD 00002033	0.002339962	C22H18O11
dexamethasone CTD 00005779	5.86E-15	C22H29FO5
curcumin CTD 00000663	5.77E-13	C21H20O6
troglitazone CTD 00002415	2.62E-08	C24H27NO5S
genistein CTD 00007324	2.03E-04	C15H10O5

## 4 Discussion

SARS-CoV-2 is a novel coronavirus and the cause of an ongoing outbreak of COVID-19 (38, 39). As the pandemic expands, there is growing concern about the impact of COVID-19 on patients with IBD. We know that a large number of proinflammatory cytokines and chemokines are key players in the orchestra during IBD development and progression, which is equivalent to the pathological process of cytokine storm caused by overactivation of innate immunity in COVID-19 (40). Thus, SARS-CoV-2 infection may accelerate the progression of IBD. Nevertheless, clinical data from the real world is lacking and there is little evidence illustrating the interactions between two diseases.

In this study, we developed a network-based and single cell atlas-based approach to investigate the expression profiles of DEGs from four RNA-seq datasets and two scRNA-seq datasets of IBD and COVID-19 patient, and identified potential molecular targets to identify the association between these two diseases. Here, we identified 121 common DEGs between COVID-19 and IBD, and discovered the biological functions of shared DEGs in the pathogenesis of COVID-19 and IBD. It is somewhat surprising that these common DEGs were significantly enriched in cytokine-mediated signaling pathway, neutrophil mediated immunity, and inflammatory response. Studies have shown that severe SARS-CoV-2 infection induces massive cytokine-mediated inflammation and neutrophil mediated immunity, leading to multiple clinical manifestations in patients with COVID-19 (41, 42). Meanwhile, chronic intestinal inflammation is triggered by cytokine-mediated pathways, which is one of the leading causes of IBD (43). We also conducted disease oncology enrichment analysis to predict the association of shared DEGs with different diseases. The findings showed that various diseases related to COVID-19, including obstructive lung disease, kidney disease, inflammatory bowel disease and ulcerative colitis. Studies have shown that obstructive pulmonary diseases, such as chronic obstructive pulmonary disease (COPD) and idiopathic pulmonary fibrosis (IFP), are regarded as high-risk factors for SARS-Cov-2 infection (44, 45). Concurrently, clinical studies have suggested that patients with COVID-19 were accompanied by clinical phenomena such as acute renal injury, proteinuria, and hematuria, which increases the mortality of patients with visceral diseases (46, 47). Angiotensin converting enzyme 2, which is used by coronavirus to penetrate target cells, is highly expressed in the ileum and colon terminus, especially during inflammation (11, 48). KEGG pathway enrichment analysis was the main approach to reflect an organism’s reactions through internal changes, and common DEGs were used to identify the similar pathways for COVID-19 and IBD patients. The results showed that IL-18 signaling pathway, PI_3_K-Akt signaling pathway and TNF signaling pathway; IL-17 signaling pathway, cytokine-cytokine receptor interaction, cytokine signaling in immune system and inflammatory response were significantly interacted with these common DEGs. These screened pathways are closely related to inflammatory response. Previous studies have indicated that IBD is an inflammatory disease mediated by deregulated inflammatory cytokines (49), and a large number of proinflammatory cytokines are detected in patients with lung injury caused by COVID-19 (50). Therefore, results from GO, DO and pathway enrichment analysis implied that the progression of IBD patients may be affected by SARS-CoV-2 infection, especially in the aspects of inflammatory response.

Subsequently, 121 common DEGs were carried out to construct a PPI network to understand the biological function properties of proteins and identify the hub genes. Here, 59 hub genes, identified on the basis of degree algorithm (51), might be key drug targets or biomarkers for COVID-19 and IBD, and associate with various pathological and biological mechanisms. As scRNA-seq technology can explore the distribution of genes expressions in different cell types (52), we further investigated the characteristics of 59 hub genes in immune cells of COVID-19 and IBD patients by single-cell atlas. Our data proved that there was an increase in CD14^+^ monocytes and a decrease in DCs and CD16^+^ monocytes cells in patients infected with SARS-CoV-2, compared with healthy controls. Of note, 38 of 59 hub genes were broadly enriched in CD14^+^ monocytes compared to other cell types., which implied that CD14^+^ monocytes were the main source of cytokine storms in COVID-19, consistent with previous studies (53).Combined with the results from transcriptome analysis, we can infer that these hub genes such as *Il1b, S100a, Il1rn* and other inflammatory factors may be the main contributors to the cytokine storm in COVID-19 patients.

Interestingly, the immune characteristics of 38 hub genes analyzed in COVID-19 showed similar results in the scRNA-seq atlas of IBD (including CD and UC). Inflammatory macrophages (IMacrophages, or M1 macrophages) were more abundant in CD and UC patients, while tumor-related macrophages (TRMacrophages, or M2 macrophages) were significantly decreased. The expression of these inflammation-related hub genes, referred to as IRGs, in different myeloid subpopulations showed that most genes were highly expressed in IMacrophages. In addition, these IRGs were highly expressed in UC and CD patients compared to the healthy controls. And we also discovered the difference in the expression profiles of IRGs between UC and DC patients. The expression of *Ido1, Ccl2, Il1rn, Tlr8, Cxcl1, Bcl2a1, Plau, Fpr2, Ptgs2* and *Tlr2* was significantly increased in CD patients, while others were more abundant in UC patients. Previous studies have shown that M1 macrophages are significantly enriched in IBD patients and secret substantial inflammatory cytokines to promote the progression of IBD patients by activating the inflammatory response (54). Furthermore, epidemiological studies have shown a greater predominance and severity of CD in women than in men, while the results were opposite for UC (55). Our results showed that female CD patients expressed higher level of IRGs than male CD patients, while male UC patients expressed higher level of IRGs than female CD patients. We also analyzed the differences in the expression of IRGs before and after TNF-α inhibitor therapy in IBD patients. The results demonstrated that the expression of most IRGs decreased significantly after treatment for IBD, suggesting that these IRGs, which were also highly expressed in COVID-19, may promote the progression of IBD through inflammatory response. Besides, these IRGs might display unique features and confer different risk factors in IBD patients of different genders.

The interaction analysis of TFs-genes and miRNAs-genes was performed to reveal the regulators of the transcription and expression of common DEGs. A total of 59 TFs and 69 miRNAs regulatory signals were identified and strongly interacted with common DEGs. For example, *Stat3*, *Yy1, Gata2*, are associated with various types of IBD diseases (56–58). Furthermore, some miRNAs, such as, miR-335-5p, miR-22-3p and miR-26b-5p, were involved in pulmonary disease, such as COPD and lung cancer (37, 59, 60).

As the pandemic expands, there is an urgent need to identify new medications to treat SARS-CoV-2 infection. Here, some potential drugs and compounds were identified for these two diseases, such as, estradiol, dexamethasone, curcumin, and Epigallocatechin gallate. Studies found that estradiol can be used for anti-inflammation, remodelling immune cell capacity, and improving the severity of colitis (61, 62). Intravenous dexamethasone combined with standard therapy can improve the condition of patients with COVID-19 and reduce lung injury in these patients (63). Curcumin can be used in anti-inflammation and anti-virus process (64), as well as regulating the immune balance in inflammatory bowel disease (65). Low dose Epigallocatechin Gallate alleviates colitis by reducing inflammatory cells and cytokines (66). Therefore, these chemical compounds can be candidate drugs for the treatment of COVID-19 and IBD.

In conclusion, our work is the first to explore the potential interaction between COVID-19 and IBD-associated gene expression using transcriptome and single cell sequencing data. Our findings identified the promotion of COVID-19 to the IBD severity by excessive cytokine storm, and may help with the understanding and medication management in IBD patients infected with SARS-CoV-2.

## Data availability statement

The datasets presented in this study can be found in online repositories. The names of the repository/repositories and accession number(s) can be found in the article/**Supplementary Material**.

## Author contributions

JW, ZC, JC and XZ supervised the project, designed, and led out the experiments of this study. CZ, ZM and XN conducted the experiments and data analysis. WW prepared all the figures and tables. CZ, ZM and XN drafted the manuscript. All authors contributed to the article and approved the submitted version.
