# Supplementary material for: Comprehensive analysis to identify the influences of SARS-CoV-2 infections to inflammatory bowel disease
Source: Front Immunol. 2023 Feb 3;14:1024041. doi: 10.3389/fimmu.2023.1024041 (PMC9936160; doi:10.3389/fimmu.2023.1024041)
Supplement: Supplementary file 1 [file DataSheet_1.docx]

Supplementary Material

**
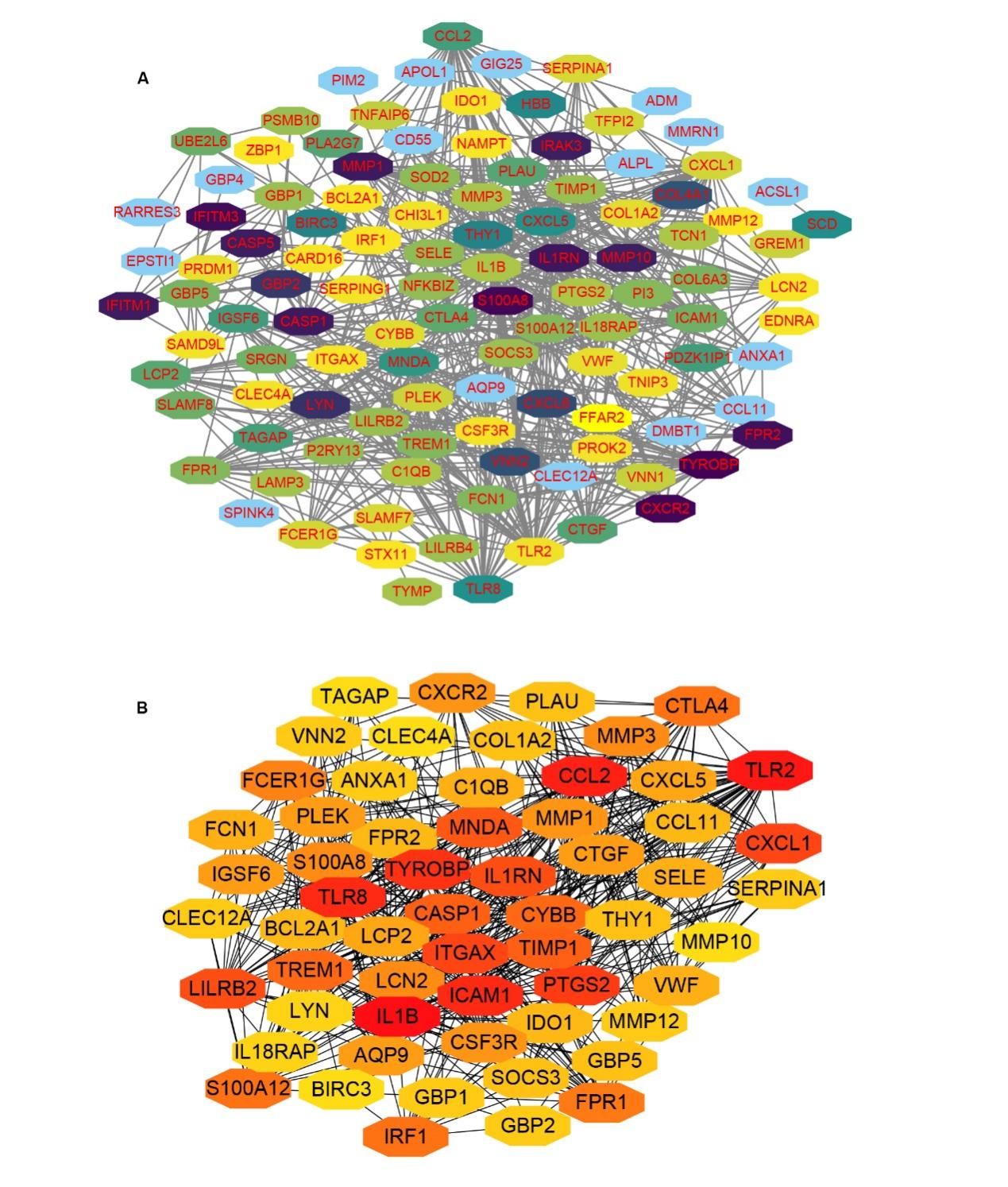
**

**Supplementary Figure 1.** **PPI network of common DEGs among COVID-19 and IBD.** (A) The PPI network of common DEGs from the STRING database. (B) The PPI network of the hub genes between COVID-19 and IBD.

**
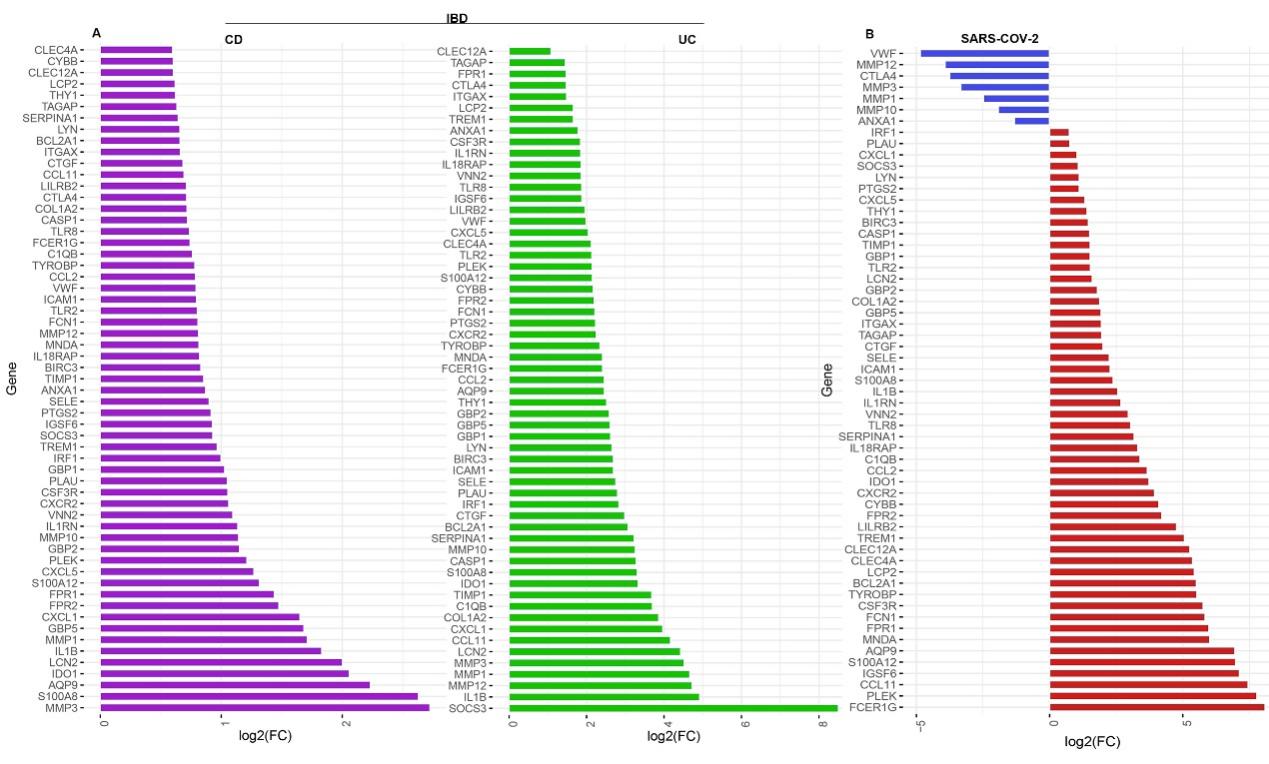
**

**Supplementary Figure 2.** **The histogram of the expression of hub genes between COVID-19 and IBD.**

**
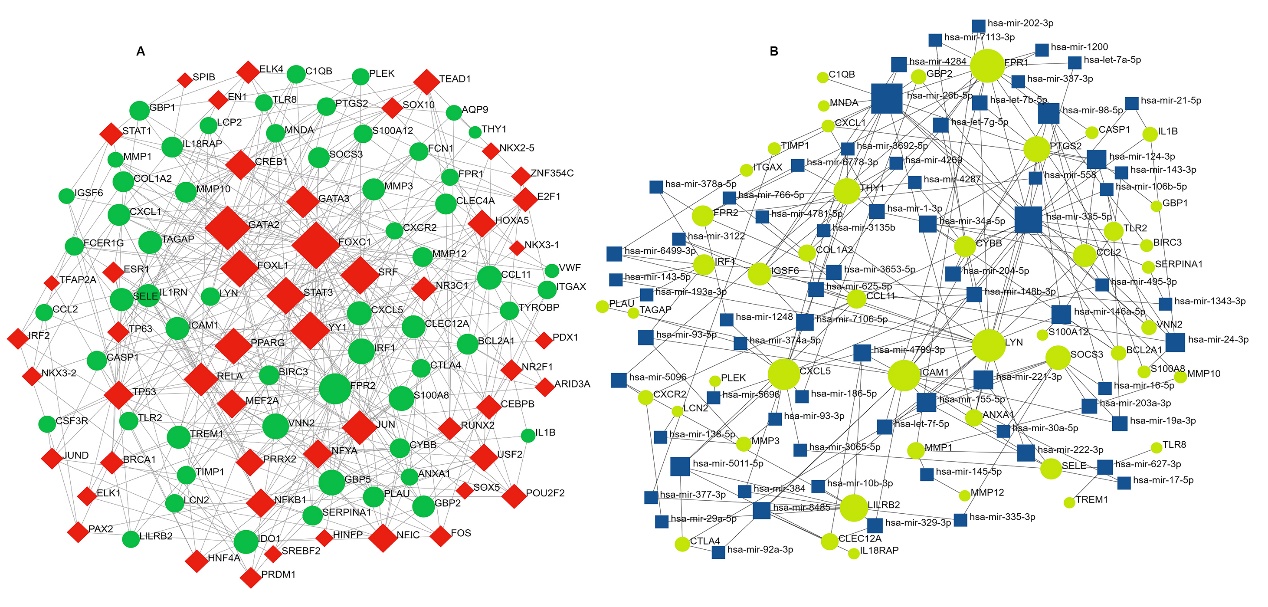
**

**Supplementary Figure 3.** **The regulatory interaction network of TFs-DEGs and miRNA-DEGs obtained from the Network Analyst.** (A) The Network Analyst created a regulatory interaction network of TFs-DEGs. Red square nodes represent TFs. Green circular nodes represent genes, which interact with TFs. (B) The Network Analyst created a regulatory interaction network of miRNA-DEGs. Blue square nodes represent miRNAs. Green circular nodes represent genes, which interact with miRNAs.


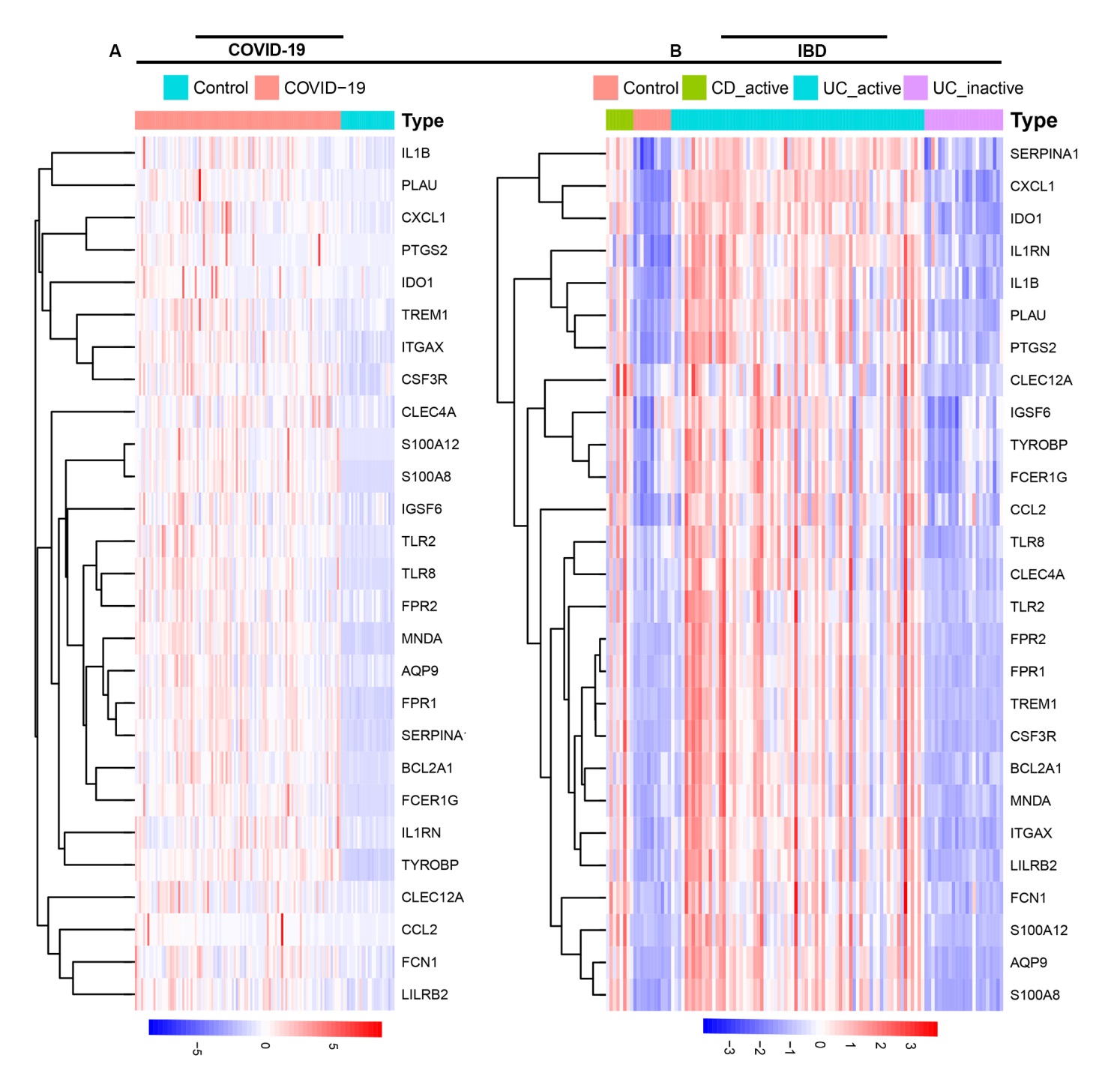


Supplementary Figure 4. Expression of 27 IRGs in the validated datasets of COVID-19 and IBD patients.

1. The heatmap of 27 IRGs expression between COVID-19 patients and healthy controls.(B) The heatmap of 27 IRGs expression among active CD patients, active UC patients, inactive UC patients and healthy controls.

**Supplementary Table 1. Details of the 121 DEGs between COVID-19 and IBD patients.**

**Supplementary Table 2. The top 15 enriched GO terms of the common DEGs.**

**Supplementary Table 3. The top 40 enriched DO terms of the common DEGs.**

**Supplementary Table 4. Enriched WikiPathways and KEGG 2019 human Pathways associated with the common DEGs.**

**Supplementary Table 5. Enriched Biocarta pathways and Hallmark Pathways associated with the common DEGs.**

**Supplementary Table 6. Degree scores of the top 59 DEGs as hub genes.**

**Supplementary Table 7. The top 10 enriched GO and DO terms of the common IRGs.**

**Supplementary Table 8. Enriched WikiPathways, Biocarta pathways , Hallmark Pathways and KEGG 2019 human Pathways associated with the common IRGs.**
